# Quality Assurance in Cervical Cancer Screening: Evaluation of Sample Adequacy in HPV DNA Testing

**DOI:** 10.1002/jmv.70482

**Published:** 2025-07-02

**Authors:** M. d'Avenia, F. Dell'Anno, M. Martinelli, L. Santomauro, R. C. Njoku, L.S. Arroyo Mühr, M. Iacobellis, C. E. Cocuzza

**Affiliations:** ^1^ School of Medicine and Surgery University of Milano‐Bicocca Milan Italy; ^2^ UOSVD Cytopathology and Screening Laboratory, ASL BARI Bari Italy; ^3^ National Reference Center of Veterinary and Comparative Oncology (CEROVEC), Istituto Zooprofilattico Sperimentale del Piemonte, Liguria e Valle d'Aosta Genoa Italy; ^4^ Department of Clinical Science, Intervention and Technology (CLINTEC), Karolinska Institutet Center for Cervical Cancer Elimination Stockholm Sweden

**Keywords:** cervical cancer screening, HPV‐DNA test quality assurance (QA), sample adequacy, sample cellularity

## Abstract

In HPV‐primary screening, sample quality significantly influences test accuracy. Unlike cytology‐based screening, no consensus guidelines presently exist for sample quality assessment in HPV testing. This study aims to evaluate the impact of sample cellularity on HPV testing. A total of 37 592 liquid‐based cytology (LBC) samples from women undergoing HPV‐primary screening (aged 30–64, median 48; IQR: 40–56 years) were analyzed using Cobas 4800 HPV Test (Roche). Sample adequacy was assessed by the assay's β‐globin internal control and by an independent quantitative cellularity assessment (OncoPredict HPV, Hiantis). HPV positivity rates (PR) were stratified according to β‐globin Ct values. Among the analyzed samples, 50.0%, 47.1%, 2.3%, and 0.6% had β‐globin Ct values of ≤ 28, > 28 to ≤ 32, > 32 to ≤ 34, and > 34, respectively. Overall HPV‐PR was 7.7% (2891/37 592). PR reached 9.7% in samples with β‐globin ≤ 28 Ct (1820/18 801), decreasing markedly to 1.4% for β‐globin > 34 Ct (3/214), (*p* < 0.001). Quantitative analysis showed that Cobas 4800 β‐globin Ct = 34 corresponds to approximately 1.5 × 10^3 nucleated cells/reaction. A subset of 195 HPV‐negative samples with β‐globin Ct ≥ 34 was evaluated by liquid based cytology (LBC): 19% had inadequate cellularity according to LBC guidelines, 8% were ≥ ASC‐US and 73% NILMs. 65% of adequate LBC showed cellular atrophy. These findings emphasize the importance of assessing cellularity in HPV‐screening to avoid potentially false‐negative results due to inadequate samples. Future research should focus on establishing standardized cellularity thresholds to improve screening accuracy.

**Abbreviations**
ASC‐H: atypical squamous cells‐high‐grade cannot be excluded
ASC‐US: atypical squamous cells of undetermined significance
CC: cervical cancer
CCR5: *C‐C chemokine receptor type 5*
CCS: cervical cancer screening
CEL: Local Ethic Committee
CI: confidence interval
CIN: cervical intraepithelial neoplasia
Ct: cycles threshold
EU: European Union
GPSC: Good Performance Screening Centers
HPV: human papillomavirus
HR: high risk
H‐SIL: high‐grade squamous intraepithelial lesion
IC: internal control
IFU: instruction for use
LBC: liquid‐based cytology
l‐SIL: low‐grade squamous intraepithelial lesion
MPSC: Medium Performance Screening Centers
NILM: negative for intraepithelial lesion or malignancy
OR: odds ratio
PCR: polymerase chain reaction
PPSC: Poor Performance Screening Centers
PR: positive rate
QC: quality control
RXN: reaction
SC: Screening Clinic
TPSC: Top Performance Screening Centers

## 1 Background

Cervical cancer (CC) is determined by persistent infection with high‐risk (HR) human papillomavirus (HPV) genotypes. The aim of HPV‐primary screening is the prevention of CC in asymptomatic women using evidence‐based protocols [1, 2, 3], balancing benefits and harms on the basis of the clinical sensitivity and specificity of validated tests. HPV‐primary screening algorithms [1, 2] are presently recommended by European Guidelines, as they have been shown to markedly improve the effectiveness of CC prevention. Despite the improved clinical sensitivity of HPV screening (92.6%) versus Pap‐test (62.8%) [3], 5.5%–16.9% of CCs are still reported as HPV‐negative, especially in women over the age of 50 years [4, 5, 6, 7]. Recent studies have shown that HR‐HPV negative LBC (Liquid Based Cytology) and Formalin‐Fixed Paraffin‐Embedded tissue samples represent 5%–7.8% of all histologically confirmed CCs [8], therefore only few rare CCs are truly HPV‐negative [8, 9, 10, 11, 12, 13] even after testing with highly sensitive assays such as Next Generation Sequencing (NGS) [4, 8].

Clinical validation studies of new HPV assays [14, 15] have focused on the clinical sensitivity and specificity of HPV targets' cut‐offs for the detection of CIN2+ lesions [16] but less attention has been placed on the assessment of sample adequacy.

Sample quality is well defined for LBC by the Bethesda System which classifies samples as “satisfactory” based on the assessment of samples' cellularity and the presence of transformation zone components [17, 18, 19, 20]. In cervical cytology, a minimum of 5000–8000 squamous epithelial cells per slide is recommended [18, 19, 21], as inadequate cellularity can lead to false negative results.

Most commercially available HPV assays are qualitative and arbitrarily estimate the amount of collected cells by applying a Cycle threshold (Ct) cut‐off for the amplification of a human internal control (IC) target, as a measure of sample adequacy. Although sample adequacy is essential for the quality assurance of HPV DNA molecular testing in CC prevention, this parameter has not been strictly evaluated, nor a minimum required number of cells has been defined to improve confidence in “HPV‐negative” results, as in the case of cervical cytology. Low‐quality samples can in fact result in HPV‐DNA false negative results due to low viral loads or other pre‐analytical issues [4, 8, 9].

The inclusion of an appropriate Sample Adequacy Control is critical in the quality assurance of PCR testing, ensuring the presence of sufficient, good quality human nucleic acids in the reaction [22, 23]. The absence of IC in some HPV assays [24, 25, 26, 27] may increase false negatives, as issues in sample collection or preanalytical processing can compromise results [8, 28]. Currently, a negative IC result indicates an invalid test outcome [28, 29] in acellular samples, however, detectable IC amplification at high PCR Ct values, within the indicated assays’ manufacturer's cut‐offs, does not always guarantee the presence of sufficient biological material. Quantitative cellularity assessment or appropriately “selected” Ct values can strengthen confidence in HPV negative results [22, 23].

The aim of this study was to determine whether the cellularity of clinical samples can influence HPV positivity rates in cervical cancer screening (CCS) programs.

## 2 Materials and Methods

### 2.1 Sample Collection and Study Population

In Apulia (Italy) the CCS program transitioned to primary HPV‐based algorithm at the end of 2022. This study analyzed 37 592 samples collected from women living in Bari metropolitan area, aged 30 to 64 years (median age 48 years; IQR: 40–56) and attending the first round of HPV‐based primary CCS, from January to December 2023. Cervical samples were collected using Cervex‐Brush (Rovers) and 20 mL PreservCyt Collection Medium (Hologic), according to manufacturer's instructions, in 45 different public CC screening clinics (SCs) by professionally trained midwives. All samples were subsequently centralized and analyzed in a laboratory performing both molecular HPV testing and cytological triage, within 1 week from sample collection.

### 2.2 HPV‐DNA Assay

Samples were analyzed using the Cobas 4800 HPV Test (Roche): this test targets the L1 gene [29] and reports individually HPV16, HPV18 and a pool of 12 additional HR‐HPV types. Human β‐globin gene is amplified as IC, confirming the qualitative presence of human nucleated cells in the sample and evaluating any potential PCR inhibition.

Testing was performed strictly following the assay's “European Instructions for Use” (EU IFU), by loading the primary collection tube onto the Cobas®x480 immediately after vortexing and decapping. From 400 μL starting volume, DNA was extracted and purified in 150 μL of elution buffer from which 25 μL was used for PCR and HPV detection.

The clinical cut‐off values for each channel (Ct < 40.5, 40, and 40 for HPV16, HPV18, and HPV “other” HR, respectively) were previously established by Rao et al. [29] to achieve the required clinical sensitivity and specificity for the detection of CIN2+ lesions. The manufacturer's IC cut‐off value for β‐globin is set at Ct 40.

### 2.3 Liquid‐Based Cytology

LBC was performed using the ThinPrep 2000 Processor (Hologic), following the manufacturer's instructions. Cytology results were reported according to the Bethesda System guidelines. LBC results were classified as “negative” if cytology was Negative for Intraepithelial Lesions or Malignancy, (NILM); as “inadequate” or “non‐diagnostic” in cases such as low cellularity (< 5000 cells/slide), excessive blood, mucus, or inflammatory cells, presence of noncellular amorphous material or “amorphous debris”; any other result was considered positive. Among NILM samples, those showing atrophy were annotated [17].

As part of the local primary HPV‐based screening algorithm, LBC was performed for the triage of HPV‐positive samples. For the present study, LBC was also performed on a subset of 195 HPV‐negative samples with β‐globin Ct ≥ 34 following which HPV‐negative women with ASC‐US+ or inadequate cytology were recalled for screening.

### 2.4 Testing of Random HPV‐Negative Samples With OncoPredict HPV QC Module

The Quality Control (QC) module of the OncoPredict HPV Quantitative Typing (QT) kit (Hiantis SRL, Milan, Italy) was used on the CFX96 Real‐Time PCR Detection System (Bio‐Rad, Hercules, CA, USA) using 5 µL of the same DNA eluate of 125 random HPV‐negative samples extracted and tested by the Cobas x480 instrument (24 < β‐globin Ct < 40). The QC module accurately quantifies the number of human cells in the sample through quantitative CCR5 gene Real‐time PCR reaction and assesses potential PCR inhibition through the separate amplification of a synthetic control target [30, 31]. Standard curves for CCR5 quantification were constructed based on the Ct values of four quantitative standards provided by the kit which were run in triplicate.

### 2.5 Evaluation of PreservCyt Volume Required for LBC Setup

The volume of 110 ThinPrep containers with IC amplified at varying Ct values was assessed, before and after cytology preparation.

### 2.6 Statistical Analysis

Data were generated by Cobas 4800 Software v2.2.0. The data archived were imported into Cobas 4800 ArchiveViewer3.0 Software. Selected runs were exported into Microsoft Excel files, and data were consolidated into a single Excel file with barcode, HPV HR Ct, HPV‐16 Ct, HPV18 Ct and β‐globin Ct. Age and data of women screened at different participating SCs associated with barcodes were exported from the Laboratory Information System. A merged pseudonymized data file was generated by a Visual Basic for Applications (VBA) code. Data were evaluated for the distribution of β‐globin Ct values, age ranges and the performance of SCs in relation to HPV results. Descriptive statistics were computed and Chi‐squared (*χ*²) tests were used. A logistic regression analysis was performed to identify factors associated with the presence of HPV. Both univariate and multivariate models were applied. The dependent variable was “HPV presence” (positive/negative) and independent variables included β‐globin Ct values, the performance of SCs and women's age intervals.

β‐globin Ct values were grouped into four categories: ≤ 28 (reference), 28 < β‐globin ≤ 32, 32 < β‐globin ≤ 34 and 34 < β‐globin ≤ 40. Performances of SCs were categorized into four groups based on β‐globin ranges as indicator of sample collection quality: Top Performance SCs (TPSC), Good Performance SCs (GPSC), Medium Performance SCs (MPSC), Poor Performance SC (PPSC), (Table SI). Women's age intervals were arbitrarily divided into four groups: 30–39, 40–49, and 50–59, 60–64 years.

Univariate logistic regression was first conducted to evaluate the association of each independent variable with HPV‐result and odds ratios (ORs) with 95% confidence intervals (CIs) were calculated. Variables with a *p*‐value < 0.05 in the univariate analysis were subsequently included in the multivariate model to adjust for potential confounders. Statistical significance was set at *p* < 0.05. All analyses were performed using STATA Statistical Software version 17.0.

## 3 Results

### 3.1 HPV Positivity Rates Related to β‐globin Ct

The results from the first round of HPV‐based primary CCS of women living in the Bari metropolitan area, showed an overall HPV positive rate (PR) of 7.7% (2891/37 592). Among the HPV positive samples, 2203 samples (76.2%) were identified as positive for “other” HR‐HPV types, 419 samples (14.5%) were positive only for HPV16, 123 (4.2%) only for HPV18, 106 (3.7%) for HPV16 and “other” HR types, 34 (1.2%) for HPV18 and “other” HR types, 2 samples (0.06%) were co‐positive for HPV16 and HPV18, and 4 (0.14%) samples showed positivity for a combination of HPV16, HPV18, and “other” HR types.

As part of the laboratory quality assurance assessment, HPV‐PR were analysed in association with β‐globin Ct values. Among the 37 592 analyzed samples, 50% (18 801), 47.1% (17 704), 2.3% (837) and 0.6% (214) had a β‐globin Ct value respectively ≤ 28, 28 < β‐globin ≤ 32, 32 < β‐globin ≤ 34, and > 34. Data showed a marked decrease in HR‐HPV PR as the β‐globin Ct values increased. Specifically, PR were highest, at 9.7%, for β‐globin ≤ 28 Ct, decreasing to 5.8% for the range 28 < Ct ≤ 32, further dropping to 1.4% in the β‐globin Ct range > 34 (Table 1). From logistic regression β‐globin Ct values showed a strong inverse association with HPV PR (*χ*² = 219.8, *p* < 0.001, Table 2). In both univariate and multivariate analyses, samples with a range 28 ˂ β‐globin ≤ 32 had significantly lower odds of HPV PR compared to the reference group (β‐globin ≤ 28), with ORs of 0.58 (95% CI: 0.53–0.63, *p* < 0.001) and 0.63 (95% CI: 0.58–0.68, *p* < 0.001), respectively. The association was even stronger for samples with 32 ≤ β‐globin < 34, which had ORs of 0.38 (95% CI: 0.27–0.57, *p* < 0.001) and 0.42 (95% CI: 0.30–0.60, *p* < 0.001). Samples with 34 ≤ β‐globin ≤ 40 range had the lowest odds of HPV‐PR, with ORs of 0.13 (95% CI: 0.04–0.41, *p* < 0.001) in the univariate model and 0.14 (95% CI: 0.05–0.45, *p* = 0.001) in the multivariate model.

**Table 1:** HPV test results sorted by β‐globin Ct‐value.

	β‐globin (Ct values)					
HPV	β‐globin ≤ 28	28 < β‐globin ≤ 32	32 < β‐globin ≤ 34	34 < β‐globin ≤ 40	Total	Chi squared *χ*² (*p*‐value)
	*N* (%)	*N* (%)	*N* (%)	*N* (%)	*N* (%)	
Negative	16 981	16.670	839	211	34 701	
	(90.3)	(94.2)	(96.1)	(98.6)	(92.3)	219.9 < 0.001
Positive	1820	1034	34	3	2891	
	(9.7)	(5.8)	(3.9)	(1.4)	(7.7)	
Total	18 801	17 704	873	214	37 592	
	(100)	(100)	(100)	(100)	(100)	

*Note:* Numbers (N) and percentages (%) of positive and negative HPV cases for each Ct group of β‐globin amplification.

**Table 2:** Association between HPV Positivity Rate (PR) and respectively β‐globin Ct values, age range and screening out‐patients' clinics (SC) grouped by estimated performance.

Variables	Univariate model (OR [IC 95%]; *p*‐value)	Multivariate model (OR [IC 95%]; *p*‐value)
β‐globin (Ct values)		
β‐globin ≤ 28	Reference	Reference
28 < β‐globin ≤ 32	0.58 [0.53–0.63]; < 0.001	0.63 [0.58–0.68]; < 0.001
32 < β‐globin ≤ 34	0.38 [0.27–0.53]; < 0.001	0.42 [0.30–0.60]; < 0.001
34 < β‐globin ≤ 40	0.13 [0.04–0.41]; < 0.001	0.14 [0.05–0.45]; 0.001
Age range		
30–39 years	2.55 [2.20–2.96]; < 0.001	2.18 [1.87–2.53]; < 0.001
40–49 years	1.70 [1.46–1.97]; < 0.001	1.50 [1.28–1.74]; < 0.001
50–59 years	1.26 [1.08–1.47]; 0.003	1.19 [1.02–1.38]; 0.03
60–64 years	Reference	Reference
Sampling Centres (SC)		
TPSC	Reference	Reference
GPSC	0.77 [0.64–0.93]; 0.006	0.81 [0.67–0.98]; 0.028
MPSC	0.69 [0.58–0.82]; < 0.001	0.76 [0.63–0.91]; 0.003
PPSC	0.79 [0.66–0.95]; 0.011	0.92 [0.77–1.11]; 0.42

*Note:* Results of logistic regression analyses (univariate and multivariate) are reported as Odds Ratio and IC95%. Top (TPSC), Good (GPSC), Medium (MPSC), Poor Performance SC (PPSC).

### 3.2 Age‐Related Sample Cellularity and HPV‐PRH

DNA yields and HPV‐PR were influenced by patients' age. Table 3 shows slight but significant differences in median age across the β‐globin Ct groups (unpaired *t*‐test *p* value < 0.0001). Generally, lower Ct values (indicating higher DNA quantity) were associated with women's younger median ages (46, IQR: 38–53). This trend may suggest that sample cellularity, as reflected by β‐globin Ct, could vary with age. However, the differences are not substantial, suggesting that age may not be the only factor influencing it. The association between age ranges and HPV‐PR was statistically significant (*χ*² = 273.0, *p* < 0.001) (Table 4), confirming previously reported data [32, 33]. In the present study, age was a significant factor in HPV‐PR: 11.5% of samples were HPV‐positive in the 30–39 yrs age group, compared to 4.8% in the 60–64 yrs age group. The 30–39 years age group had the highest odds of HPV‐PR, with ORs of 2.55 (95% CI: 2.20–2.96, *p* < 0.001) in the univariate model and 2.18 (95% CI: 1.87–2.53, *p* < 0.001) in the multivariate model. The 40–49 years group had ORs of 1.70 (95% CI: 1.46–1.97, *p* < 0.001) and 1.50 (95% CI: 1.28–1.74, *p* < 0.001) in univariate and multivariate analyses, respectively. The 50–59 years age group had lower but still significant odds, with ORs of 1.26 (95% CI: 1.08–1.47, *p* = 0.003) and 1.19 (95% CI: 1.02–1.38, *p* = 0.025) in the univariate and multivariate models, respectively, (Table 2).

**Table 3:** Median age and interquartile range (IQR) in each β‐globin Ct range.

Cellularity	Age (Median)	IQR	N	%
β‐globin ≤ 28ct				
Positive	43.0	36–51	1820	
Negative	46.0	39–53	16 981	
Total	46.0	38–53	18 801	50
28 < β‐globin ≤ 32				
Positive	47.0	39–56	1034	
Negative	51.0	43–58	16 670	
Total	51.0	43–57	17 704	47.1
32 < β‐globin ≤ 34				
Positive	52.5	42–58	34	
Negative	55.0	47–59	839	
Total	55.0	47–59	873	2.3
34 < β‐globin ≤ 40				
Positive	42.0	31–60	3	
Negative	54.0	47–59	211	
Total	54.0	47–59	214	0.6
Total				
Positive	45.0	37–53	2891	
Negative	49.0	41–56	34 701	
Grand Total	48.0	40–56	37 592	100

*Note: N* = sample count in each group. Age unpaired *T*‐Test *p* value < 0.0001 vs. β‐globin ≤ 28ct group.

**Table 4:** HPV test results sorted by age range.

	Age range					
HPV	30–39	40–49	50–59	60–64	Total	Chi squared *χ*² (*p*‐value)
	*N* (%)	*N* (%)	*N* (%)	*N* (%)	*N* (%)	
Negative	7459	10 996	11 790	4456	34 701	
	(88.54)	(92.08)	(93.99)	(95.17)	(92.3)	273.0 < 0.001
Positive	965	946	754	226	2891	
	(11.5)	(7.9)	(6.01)	(4.8)	(7.7)	
Total	17 701	18 728	931	232	37 592	
	(100)	(100)	(100)	(100)	(100)	

*Note:* Numbers (N) and percentages (%) of positive and negative cases for each age range; the *p*‐value from the *χ*² test for each group is compared to the total group.

### 3.3 Sample Collection Variability Across Screening Clinics

The study also evaluated the variation in sample cellularity across 45 different SCs in the metropolitan area of Bari, where samples were collected for HPV testing. β‐globin Ct distributions were assessed in samples collected from different SCs, and it was observed that in some clinics the samples collected showed a significantly higher cellularity compared to others as indicated by the distribution of β‐globin Ct values. Given that patient age did not significantly differ across centers (patient median age in clinics = 48, IQR: 41–56), these variations are likely to reflect differences in performing sample collection by the health professionals operating in the different SCs.

Therefore, we sorted SCs into four groups (Table SI). As shown in Table 5, some SCs had a higher percentage of samples in the β‐globin ≤ 28 Ct range as compared to others which had a greater proportion of samples with higher intervals of β‐globin Ct values (28 < β‐globin ≤ 32, 32 < β‐globin ≤ 34 and 34 < β‐globin ≤ 40), (Table SII).

**Table 5:** DNA yields grouped by β‐globin Ct ranges sorted by the performance of screening clinics (SC) involved in the study.

	Performance of sampling centres (SC)					
β‐globin (Ct values)	TPSC	GPSC	MPSC	PPSC	Total	Chi squared *χ*² (*p*‐value)
	*N* (%)	*N* (%)	*N* (%)	*N* (%)	*N* (%)	
β‐globin ≤ 28	857	4036	9241	4667	18 801	
	(57.4)	(55.4)	(51.9)	(42.4)	(50)	881.6 < 0.001
28 < β‐globin ≤ 32	609	3221	8192	5682	17 704	
	(40.8)	(44.2)	(46)	(51.6)	(47.1)	
32 < β‐globin ≤ 34	20	23	293	537	873	
	(1.8)	(0.4)	(1.6)	(4.8)	(2.3)	
34 < β‐globin < 40	7	0	74	133	214	
	(0.5)	(0.0)	(0.4)	(1.2)	(0.6)	
Total	1493	7280	17 800	11 019	37 592	
	(100)	(100)	(100)	(100)	(100)	

*Note:* The *p*‐value from the *χ*² test for each group is compared to the total group. Screening Clinics (SCs) were sorted in four groups on the basis of cellularity of the samples collected: Top (TPSC), Good (GPSC), Medium (MPSC), Poor Performance SC (PPSC).

The correlation between performance of SCs and HPV PR was assessed with a regression model (Table 2). GPSC showed reduced odds of HPV‐PR compared to TPSC in both univariate (OR = 0.77, 95% CI: 0.64–0.93, *p* = 0.006) and multivariate analyses (OR = 0.81, 95% CI: 0.67–0.98, *p* = 0.028). MPSC had ORs of 0.69 (95% CI: 0.58–0.82, *p* < 0.001) and 0.76 (95% CI: 0.63–0.91, *p* = 0.003) in univariate and multivariate models, respectively. PPSC were significantly associated with reduced odds of HPV PR only in the univariate analysis (OR = 0.79, 95% CI: 0.66–0.95, *p* = 0.011), but not in the multivariate model (OR = 0.92, 95% CI: 0.77–1.11, *p* = 0.402).

### 3.4 Correlation Between Sample Cellularity and β‐Globin Ct Values

The study further evaluated the relationship between β‐globin Ct values and a quantitative sample cellularity assessment. The results of testing 125 HPV‐negative random samples with OncoPredict HPV QC module [30, 31] underlined that higher β‐globin Ct values are due to a lower initial amount of human DNA or cells in the sample. Moreover, the PCR inhibition control included in the OncoPredict HPV QC module did not show any evidence of inhibition of the reaction among tested samples, which could account for the higher β‐globin Ct values. This is consistent with the hypothesis that β‐globin late amplification using Cobas 4800 correlates with samples with a lower cellular content, rather than resulting from PCR inhibition issues.

**Figure 1:**
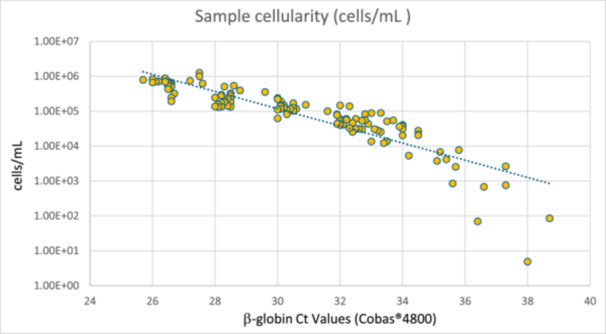
Sample cellularity/mL vs β‐globin Ct values assessed by CCR5‐qPCR assay.

Graph in Figure 1 plots the number of cells per mL, as a function of β‐globin Ct values, calculated by CCR5 standard curve, in 125 samples with β‐globin Ct ranging from 25 to 40.

Quantification showed cellularity in the LBC sample and in Cobas 4800 PCR reaction mix respectively of about 2 × 10^5 cells/mL and 1.5 × 10^4 cells/reaction (rxn) at a β‐globin Ct of 28, 6 × 10^4 cells/mL and 4 × 10^3 cells/rxn at a β‐globin Ct of 32, 2.5 × 10^4 cells/mL and 1.5 × 10^3 cells/rxn at a β‐globin Ct of 34, showing a significant drop in nucleated cells with increasing Ct. At a Ct of 39, the cellularity reached the lowest measurable levels, close to 10 cells/mL and 1 equivalent genome/rxn, indicating that very few nucleated cells are present in the tested sample, with HPV DNA test results for these samples being reported by Cobas 4800 software as “valid” and “HPV‐negative”. Moreover, IC Ct value reflects the overall number of nucleated cells in the sample, including inflammatory cells and not just the squamous epithelial cells (the primary target for HPV infection [34]), which means that Ct values represent total nucleated cell content rather than solely the target cell type for HPV.

### 3.5 Cytology and Adequacy of Samples With Late β‐globin Amplification

In a subset of 195 samples with β‐globin ≥ 34 Ct, LBC slides were prepared to assess sample adequacy. Cytology revealed that 19% (37/195) of these samples exhibited inadequate cellularity, with less than 5000 cells per slide, 8% were positive for abnormal cytology (16/195: 7 ASC‐US, 7 L‐SIL, 1 ASC‐H, 1 H‐SIL), and 65% showed normal cytology with marked atrophy, suggesting that atrophic changes in the cervical epithelium may contribute to the reduced cellular yield. The remaining samples (8%) were NILMs but not atrophic.

Within this subset of samples, those found to be HPV‐negative with inadequate cytology or positive for ≥ ASC‐US were reported as “inadequate”, and women were invited to repeat the test. Among 16 samples with positive cytology, 4 were HPV positive at the recall visit, 4 missed the recall, 8 were HPV negative (2 with β‐globin > 32 Ct). Among inadequate samples (*N* = 37), 12 women missed the recall, 3 were inadequate on repeated testing and 22 were HPV‐negative (14 with β‐globin > 32 Ct). These further emphasize the need for robust sample collection techniques to avoid missing clinically significant infections and need for recalling systems, [of note that 16/53 (30.2%) missed the recall visit].

Among the 2891 HPV‐positive samples, only four women (0.13%) had inadequate LBC results, due both to insufficient epithelial cervical squamous cells and excessive blood contamination. The β‐globin Ct values in these samples ranged from 28.2 to 30.6 (mean Ct value 29.7). These IC Ct values, all below 34, could potentially be explained by an increased number of white blood cells (WBCs) in the sample, as the β‐globin gene is present in all human nucleated cells.

**Figure 2:**
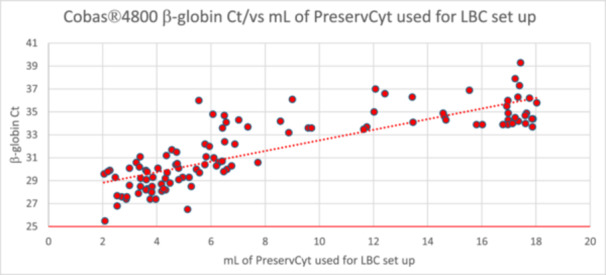
PreservCyt (mL) aspirated for LBC setup by ThinPrep 2000 vs PCR β‐globin Ct.

Moreover, we observed that among samples with β‐globin late amplification, very little PreservCyt remained in the ThinPrep vial after LBC preparation. To investigate this further and to assess potential differences in residual volume, we measured the amount of PreservCyt in 110 ThinPrep vials, with samples at varying β‐globin Ct values, before and after cytology preparation. As shown in Figure 2, there is a strong correlation between the volume aspirated for cytology preparation and the β‐globin Ct. This confirms that when the IC Ct is higher, indicating a lower concentration of cells/mL in the sample, the instrument requires to aspirate more liquid to create an adequate LBC slide.

## 4 Discussion

HPV DNA tests validated for CCS, adhering to Meijer [14] and VALGENT [15] criteria, focus on achieving appropriate clinical sensitivity and specificity for the detection of CIN2+ lesions, as assessed by HR‐HPV assays' specific cut‐offs. This approach allows to discriminate clinically relevant from transient HR‐HPV infections, the latter usually associated with low viral loads, so avoiding potentially overtreatment. However, HPV DNA target cut‐offs can also be influenced by sample cellularity, affected by the intrinsic variability associated with collection from a mucosal surface, the use of different devices, volumes and collection media as well as being operator‐dependant.

This cross‐sectional study based on a screening population of 37 592 women investigated the association of HR‐HPV positivity with β‐globin Ct values. Interestingly, decreasing HR‐HPV PR were observed in samples with increasing β‐globin values, dropping from 9.7% in samples with β‐globin Ct ≤ 28% to 1.4% in samples with β‐globin Ct > 34 (*p* < 0.001). Further investigations suggested that cervical samples containing not less than 2.5 × 10^4 cells/mL (β‐globin ≤ 34 Ct) could be considered acceptable for screening purpose. The preliminary results of this study therefore underline the potential influence of sample adequacy in the performance of HPV‐primary screening programs.

While our results emphasize the importance of assessing sample cellularity in HPV molecular testing, it is difficult to establish a common Ct value cut‐off for the different molecular assays as they are based on different human gene targets. Further studies are needed to define standardized cellularity and Ct thresholds for the human targets of specific HPV assays. Moreover, as different HPV assays require different starting volumes for DNA extraction, elution and amplification, a specific sample quality assessment study is required to determine the acceptable cellular content required for each assay. Commercial HPV assays need to be assessed using the different pre‐analytic and analytic protocols used in the clinical validation studies, which can influence the IC Ct values [16, 21, 22, 23, 29].

In the present study only 50% of all cervical samples achieved β‐globin amplification within a Ct of 28, which is in contrast with the results reported from another HPV‐primary screening program in Norway [35] where all samples run on Cobas 4800 had an IC Ct value ≤ 28. This difference could be explained by the different pre‐analytic procedure used in Norway, which involved vortexing and immediate aliquoting of 1.25 mL of sample into analysis tubes, whilst in our screening laboratory primary sample vials (20 mL) were loaded directly into the instrument, following preliminary vortexing and decapping as indicated in Roche European IFUs. The higher sample volume and longer time interval, from vortexing to sample pipetting and analysis, may also account for the observed differences in the IC Ct values.

Although HPV‐based CCS has been shown to be highly effective, this study underlines the importance of implementing quality assurance measures in both the preanalytical and analytical steps.

Cellularity in cervical samples could be influenced by several factors, such as:

1. Non‐adherence to Standard Operating Procedures for cervical sample collection [36, 37, 38]: such as improper rotation of the brush, which can result in insufficient cellular material being transferred to the sample resuspension volume [38]. In this context it is worth investigating whether sample collection performance may depend on the type of collection device, brush, or swab, used.
2. Pre‐analytical Processing Issues:

− If the brush, used for sample collection, is not adequately and quickly rinsed in the solution through proper bristle opening and mixing [36, 37], many cells may remain on the device, leading to lower sample's cellularity.
− Inadequate vortexing may cause cells to remain clumped or settle at the bottom of the tube, rather than being evenly suspended.
− A delay between vortexing and sample processing can exacerbate this problem, as cells may precipitate before the test system aspirates the required volume from the meniscus of the primary vial after loading the sample on the assay's liquid handler.

3. Test IC target: different assays have different target genes and/or sequences within the gene target, which may be present in multiple and/or variable number of copies within human cells, influencing sample adequacy assessment and cellular thresholds.

It is possible to improve CCS by adequate sample collection and by improved reliability of HPV‐primary testing by addressing factors such as improved training of health operators on the procedure of sample collection and on performing pre‐analytical and analytical quality controls during sample processing and testing.

Moreover, among the causes of poor sample cellularity, patient's age and hormonal status must be taken in consideration. In older or postmenopausal and postpartum patients, the cervix may exhibit mucosal atrophic changes. Atrophy often leads to a reduced cellular yield and the formation of cellular clumps, which can negatively impact on the quality and quantity of biological material available for analysis. The literature highlights an age‐related decline in test sensitivity due to sampling challenges with reports pointing to an elevated risk of CIN2+ in older women with ASCUS but HPV‐negative [39, 40]. Although CC is more frequently diagnosed in younger women, due to routine screening, the median age at diagnosis remains 50 years. Women over 65 years account for over 20% of new CC cases and 37% of CC deaths in the United States [40], emphasizing the need for improved quality assurance measures to enhance screening effectiveness in older populations.

Despite these challenges, LBC systems, like ThinPrep 2000, partially mitigate these issues by aspirating larger volumes of medium (ranging from 2 to 18 mL depending on cellular concentration) and transferring cells onto a slide for optimal microscopic evaluation. This flexibility ensures the presence of a sufficient number of cells for cytological examination. Additionally, LBC protocols include established quality assurance benchmarks based on the number of cells, which provide a reference for sample adequacy and enhance reliability in cytological assessment as previously discussed [17, 18, 19, 20, 21]. In contrast, HPV testing currently lacks standardized quality assurance benchmarks for cellularity. These findings do not define specific cellularity thresholds applicable to all settings, and additional research is required to determine optimal cut‐off values. Moreover, standard workflows use a fixed starting sample volume, typically 0.2–0.4 mL of the resuspended cervical sample, used for DNA extraction, irrespective of the starting sample cellularity. The possibility to concentrate samples should be investigated although less feasible in high throughput screening laboratories. Another possible solution could be to report for HPV‐negative results, an indication of sample cellularity, categorizing cellular quantity as good, fair, or poor. Moreover, providing midwives feedback on their performance in sample collection could serve as an incentive for improvement.

In screening programs based on self‐collected samples, ensuring adequate sample cellularity is crucial for quality assurance and should be taken into consideration. Self‐sampling studies have shown that some women prefer clinician‐collected samples due to concerns about their ability to perform self‐collection correctly [41], highlighting the need for confidence‐building measures and quality assurance strategies.

In conclusion, future clinical studies are necessary to establish shared guidelines for minimum sample cellularity in HPV molecular testing. Such research would help define optimal IC cut‐off values across different diagnostic systems and sample collection methods, improving confidence in HPV‐negative results in cervical cancer screening programs.

## Author Contributions

Conceptualization, writing, data curation, and editing: Morena d'Avenia, Clementina E. Cocuzza. Molecular biology experiments: Morena d'Avenia, Njoku Chinyere Ruth. Cytology evaluation: Loredana Santomauro, Michela Iacobellis. Data collection: Morena d'Avenia, Njoku Chinyere R. Statistical Analysis, Tables and Figures: Filippo Dell'Anno, Morena d'Avenia. Writing review: Clementina Cocuzza, Morena d'Avenia, Marianna Martinelli, L.S. Arroyo Mühr, Filippo Dell'Anno. Supervision and funding acquisition: Michela Iacobellis, Clementina Cocuzza. All authors have read and agreed to the published version of the manuscript.

## Ethics Statement

The study received ethical approval from the Local Ethics Committee (Ref. 7438/CEL/2023, addendum 1809/CEL/2024).

## Consent

Women involved in the observational study gave written consent.

## Conflicts of Interest

C. E. C. institution has received research grants and/or gratis consumables from Beckton Dickinson, Copan Italia, Seegene, Novosanis and Fujirebio. C. E. C. has received speaker honoraria and/or travel funds from Seegene, Beckton Dickinson, Copan Italia. C. E. C. is a minority shareholder of Hiantis Srl.

## Supporting information

Supplementary Table I.

Supplementary table II.

## Data Availability

The data that support the findings of this study are available from the corresponding author upon reasonable request.
