# Supplementary material for: Quality Assurance in Cervical Cancer Screening: Evaluation of Sample Adequacy in HPV DNA Testing
Source: J Med Virol. 2025 Jul 2;97(7):e70482. doi: 10.1002/jmv.70482 (PMC12216795; doi:10.1002/jmv.70482)
Supplement: Supplementary file 1 — Supplementary Table I. [file JMV-97-e70482-s001.pdf]

Sampling centres (Screening Out-patients' clinic)		Samples N sorted by BG Ct group				Sample % in each BG Ct group				Age Mean	N
		BG<28ct	28<BG<32Ct	32<BG<34Ct	BG>34Ct	BG<28ct	28<BG<32Ct	32<BG<34Ct	BG>34Ct		
HOSPITAL DVN	TPSC	3				100	0	0	0	50,7	3
HOSPITAL CRT	TPSC	3	3			50	50	0	0	45,7	6
HOSPITAL MNP	TPSC	6	1			85,7	14,3	0	0	50,9	7
CNTR1	TPSC	16	9	1	1	59,3	33,3	3,7	3,7	46,1	27
HOSPITAL CNV	TPSC	25	24	3		48,1	46,2	5,8	0	50,1	52
HOSPITAL GRM	TPSC	35	25			58,3	41,7	0	0	47	60
PF SP	TPSC	39	23	2		60,9	35,9	3,1	0	42,5	64
CF31	PPSC	63	60	4	3	48,5	46,2	3,1	2,3	45,4	130
PF T	TPSC	76	68	5	3	50	44,7	3,3	2	43,4	152
CF20	GPSC	208	164			55,9	44,1	0	0	47,1	372
CF9	PPSC	198	200	26	10	45,6	46,1	6	2,3	48,9	434
HOSPITAL LTM	TPSC	331	156	4	3	67	31,6	0,8	0,6	43,2	494
CF3	PPSC	163	281	46	11	32,5	56,1	9,2	2,2	48	501
CF4	MPSC	313	255	8	4	54	44	1,4	0,7	47,7	580
CF34	GPSC	414	193	1		68,1	31,7	0,2	0	46,5	608
HOSPITAL DLF	TPSC	323	300	5		51,4	47,8	0,8	0	47,4	628
CF35	MPSC	352	270	9	1	55,7	42,7	1,4	0,2	46,4	632
CF30	GPSC	371	282	6		56,3	42,8	0,9	0	45,9	659
CF15	MPSC	291	367	20	3	42,7	53,9	2,9	0,4	46,1	681
CF13	MPSC	378	305	14	7	53,7	43,3	2	1	47,4	704
CF16	MPSC	359	354	7		49,9	49,2	1	0	46,9	720
CF11	MPSC	340	373	16	9	46,1	50,5	2,2	1,2	49,1	738
CF28	PPSC	373	350	38	13	48,2	45,2	4,9	1,7	47,7	774
CF7	MPSC	424	344	9	2	54,4	44,2	1,2	0,3	47,3	779
CF2	PPSC	315	437	37	4	39,7	55,1	4,7	0,5	47,4	793
CF21	GPSC	492	344	3		58,6	41	0,4	0	47,7	839
CF17	MPSC	513	319	12	2	60,6	37,7	1,4	0,2	46,3	846
CF5	GPSC	453	445			50,4	49,6	0	0	46,6	898
CF6	MPSC	513	429	14		53,7	44,9	1,5	0	47,8	956
CF25	PPSC	348	580	43	1	35,8	59,7	4,4	0,1	49,4	972
CF33	MPSC	553	475	6	4	53,3	45,8	0,6	0,4	47,3	1038
CF10	GPSC	616	458	3		57,2	42,5	0,3	0	47,5	1077
CF24	MPSC	602	536	14	2	52,2	46,4	1,2	0,2	49,9	1154
CF19	MPSC	573	585	24	1	48,4	49,5	2	0,1	48,3	1183
CF22	PPSC	426	678	79	21	35,4	56,3	6,6	1,7	48,3	1204
CF18	GPSC	659	549	7		54,2	45,2	0,6	0	47,3	1215
CF29	PPSC	545	649	61	18	42,8	51	4,8	1,4	49,4	1273
CF14	MPSC	720	583	11	3	54,7	44,3	0,8	0,2	47,5	1317
CF26	PPSC	688	752	59	14	45,5	49,7	3,9	0,9	46,6	1513
CF12	GPSC	823	786	3		51,1	48,8	0,2	0	48,1	1612
CF23	MPSC	880	758	14	2	53,2	45,8	0,8	0,1	47,2	1654
CF32	PPSC	728	864	60	14	43,7	51,9	3,6	0,8	47,5	1666
CF27	PPSC	820	831	84	24	46,6	47,2	4,8	1,4	49,4	1759
CF8	MPSC	1097	979	33	11	51,7	46,2	1,6	0,5	48,8	2120
CF1	MPSC	1333	1260	82	23	49,4	46,7	3	0,9	49,6	2698
**Total**		**18801**	**17704**	**873**	**214**	**50**	**47,1**	**2,3**	**0,6**	**47,8**	**37592**

BG: β-Globin

Ct: Cycle threshold
