# Supplementary material for: Quality Assurance in Cervical Cancer Screening: Evaluation of Sample Adequacy in HPV DNA Testing
Source: J Med Virol. 2025 Jul 2;97(7):e70482. doi: 10.1002/jmv.70482 (PMC12216795; doi:10.1002/jmv.70482)
Supplement: Supplementary file 2 — Supplementary table II. [file JMV-97-e70482-s002.pdf]

Supplementary Table II.

β-globin (Ct values)	Sampling Centres (SC)	Coefficient	P>|z|	[95% Conf. Interval] Lower	[95% Conf. Interval] Upper
β-globin ≤ 28	TPSC	15.79	0.973	-899.84	931.41
	GPSC	0.02	0.96	-0.75	0.79
	MPSC	-1.25	0.001	-2.01	-0.48
	PPSC (Con)	4.81	0	4.06	5.55
28 < β-globin ≤ 32	TPSC	15.90	0.973	-899.72	931.53
	GPSC	0.24	0.545	-0.53	1.02
	MPSC	-0.71	0.068	-1.47	0.05
	PPSC (Con)	4.47	0	3.72	5.21
32 < β-globin ≤ 34	TPSC	14.38	0.975	-901.25	930.01
	GPSC	0.33	0.476	-0.57	1.22
	MPSC	0.35	0.442	-0.53	1.23
	PPSC (Con)	1.05	0.017	0.18	1.91
34 < β-globin ≤ 40 (reference)	-	-	-	-	-

To evaluate the differences in the performance of sampling centres (screening clinics (SCs)) based on the cycle threshold (Ct) values of the β-globin gene, a multinomial logistic regression analysis was conducted, with the PPSC group set as the reference category.

The multinomial logistic regression analysis revealed differences in the performance of SC across β-globin Ct value categories. When using PPSC as the reference group, notable patterns emerged.

In the β-globin ≤ 28 Ct group, MPSC showed a statistically significant negative association (Coefficient: -1.25; P = 0.001; 95% CI: -2.01, -0.48), indicating that samples with lower Ct values were less likely to be associated with this group compared to PPSC. Conversely, TPSC exhibited a high coefficient (15.79), but this result lacked statistical significance (P = 0.973), suggesting no clear evidence of a difference from PPSC. GPSC also showed no significant association in this category.

For the 28 < β-globin Ct ≤ 32 category, no statistically significant differences were observed among the groups, though MPSC demonstrated a marginal trend toward lower performance compared to PPSC (Coefficient: -0.71; P = 0.068). Similarly, in the 32 < β-globin Ct ≤ 34 group, none of the comparisons reached statistical significance, though PPSC had a significant reference coefficient (Coefficient: 1.05; P = 0.017; 95% CI: 0.18, 1.91).

This analysis highlights the significant underperformance of MMSC compared to PPSC for samples with β-globin ≤ 28 Ct. This suggests that MPSC may struggle with efficiently collecting high-quality samples, particularly those with low Ct values, which could reflect better sample quality. In contrast, TPSC consistently exhibited high coefficients across categories, though these were not statistically significant, indicating that its performance does not strongly differ from the reference group in this analysis.
